# Shaking Up Essential Tremor: Peripheral Devices and Mechanical Strategies to Reduce Tremor

**DOI:** 10.5334/tohm.930

**Published:** 2024-11-11

**Authors:** Kian Adabi, William G. Ondo

**Affiliations:** 1 Department of Neurology, Houston Methodist Hospital, Houston, TX, US; 2 Weill Cornell Medical School, Houston, TX, US

**Keywords:** essential tremor, Action tremor, non-invasive, cooling therapy, cala trio, mechanical device, transcutaneous afferent patterned stimulation, adaptive device, vibration therapy, tremor management, neurostimulation, wearable device, clinical trials, device based interventions, tremor research

## Abstract

This review discusses non-pharmacological, non-surgical interventions for action tremor, including essential tremor (ET). We review transcutaneous peripheral nerve stimulation (PNS), a variety of orthotic/mechanical devices, cooling and vibration strategies, and adaptive utensils, most of which are currently available. The PNS section discusses open loop (CALA-Trio) and closed loop systems (Felix™, NeuroAI™ and Motimove® systems). Orthotic devices which physically dampen tremor include Tremulo™, GyroGlove™, WOTAS exoskeleton, Magnetorheological Fluid-Based Exoskeleton System, Steadi-One® and Steadi-Two®, and Readi-Steady®. Adaptive devices include weighted spoons, deep cavity spoons, counter-balance utensils, and electrical actuator devices. Despite availability, most of these devices have limited to no published clinical trial data.

## Introduction

Essential tremor (ET) is a prevalent neurological disorder characterized by involuntary, rhythmic oscillation, primarily affecting the hands, although it can also involve the head, voice, and other body parts [1]. While the exact cause of ET is not fully understood, it likely involves abnormal CNS circuits, and a combination of genetic and environmental factors [2]. Treatment options aim to reduce tremor amplitude and improve quality of life. These include pharmacological treatments, such as propranolol, nadolol, primidone, topiramate, and benzodiazepines, each with its own profile of effectiveness, side effects, and limitations, especially in more severe tremor [3,4]. Botulinum toxin injections, and surgical interventions like deep brain stimulation (DBS) and focused ultrasound represent more invasive treatments.

This review will discuss peripheral nerve stimulation, mechanical devices, vibration therapy, limb cooling, and adaptive utensils designed to compensate for tremor, and improve daily functioning, some of which have shown benefit in clinical trials but most of which lack controlled data [5,6,7,8,9].

## Methods

### Literature Search Strategy

In order to capture the full scope of technology-driven treatments, including but not limited to transcutaneous peripheral nerve stimulation (PNS), orthotic and mechanical devices, cooling therapy, vibration therapy, and adaptive utensils, a detailed search strategy was implemented, combining search terms and databases to encompass both published scientific research and grey literature. Keywords included “essential tremor,” “non-pharmacological interventions”, “action tremor”, “technology-driven treatments”, “transcutaneous peripheral nerve stimulation”, “orthotic devices”, “tremor assist devices”, “cooling therapy”, “vibration therapy”, “adaptive utensils”, “peripheral nerve stimulation AND open loop”, “peripheral nerve stimulation AND closed loop”, “wearable devices AND tremor suppression”, “non-surgical tremor treatments”, “rehabilitation devices”, and “non-invasive interventions”. The search terms were combined using Boolean operators such as AND/OR to target specific areas of interest. For example, terms like “essential tremor” were combined with “non-pharmacological interventions” OR “non-invasive treatments” AND “tremor assist devices” to ensure a comprehensive search. These combinations allowed for the inclusion of both broad categories and specific interventions, such as “peripheral nerve stimulation AND open loop” and “orthotic devices AND tremor suppression,” which narrowed the focus to relevant devices.

The search necessarily extended beyond traditional databases including PubMed, Google Scholar, Scopus, Web of Science, IEEE Xplore, the Cochrane Library, ClinicalTrials.gov, and EMBASE. Recognizing the limited availability of published clinical trial data for many of these devices, we also sought information directly from manufacturers, reviewed product brochures and Web-based information (usually provided by the commercial seller), personal communications with researchers, and considered our own experience with certain devices. Therefore, we include considerable information not published in peer reviewed scientific literature. We concentrated on available devices but made some exceptions if the devices have advanced to testing in ET subjects or were conceptually unique. After conducting the initial search, we applied several filtering steps to refine the results. Articles were first filtered by relevance, focusing on those that specifically addressed non-pharmacological, non-surgical interventions for ET and related conditions. We prioritized studies that reported clinical outcomes or device efficacy in real-world settings. Articles were then categorized by study design, giving preference to clinical trials and case studies, though in many cases, due to the emerging nature of this technology, grey literature and manufacturer-provided data were also included.

## 1.Transcutaneous Peripheral Nerve Stimulation

Transcutaneous peripheral nerve stimulation (PNS) is a promising approach for managing tremor through non-invasive electrical stimulation of peripheral nerves. The definitive mechanism of action is debated and can involve afferent and/or efferent transmission [10].

The only commercially available PNS is postulated to improve tremor control via electrical stimulation to afferent pathways, typically below the motor threshold to avoid efferent muscle contractions. This strategy targets nerves in the arm where relatively superficial (radial, medial, ulnar nerve near the wrist), possibly modulating the central nervous system, and is often called Transcutaneous Afferent Patterned Stimulation (TAPS) [11].

This contrasts with devices designed to stimulate efferent nerves, and directly contract muscles, sometimes called functional electrical stimulation [8]. Studies have investigated various efferent stimulation strategies, including co-contraction and out-of-phase stimulation, to optimize effectiveness. Simultaneous antagonist co-contraction enhances joint stability (decreases elasticity) by augmenting resistance to involuntary movements [12]. Out of phase stimulation physically counteracts tremor by contracting the muscle antagonizing the involuntary movement, but requires a near perfect real time understanding of muscle activity, and precise stimulation fidelity.

The research underscores the potential of PNS in modulating tremor severity, particularly when the stimulation frequency is phase-locked and tailored to the patient’s specific tremor characteristics. Although the modulation of tremor severity at the group level has shown mixed results, individual cases in a study completed by Reis *et al*. demonstrated significant tremor suppression, highlighting the importance of patient-specific approaches [13].

### 1.1 Open Versus Closed-Loop PNS System

Peripheral stimulation device are segregated into open-loop and closed-loop designs [14]. Open-loop systems, characterized by their fixed, pre-determined stimulation settings, provide a straightforward approach by delivering consistent electrical signals regardless of the patient’s real-time tremor intensity. This lack of responsiveness to immediate changes in tremor dynamics means that while open-loop systems can be effective, their one-size-fits-all approach may not suit every patient’s fluctuating needs [15,16].

Conversely, closed-loop systems present a more adaptable approach. By leveraging feedback from the patient’s physiological signals, these systems dynamically adjust stimulation parameters, usually current.

In a single head-to-head comparison of open-loop and closed-loop peripheral nerve stimulation systems for tremor control, a closed-loop system designed by Kim *et al*. demonstrated a statistically significant reduction in dominant tremor frequency, while open-loop systems did not significantly affect tremor frequency [12]. Although the study achieved statistically significant reductions in tremor metrics, further evaluation is needed to determine the significance of frequency reduction, as tremor amplitude is more associated with functionality. Both systems effectively reduced tremor power, but closed-loop systems excelled in frequency modulation efficiency.

### 1.2 CALA Trio

**Figure 1:**
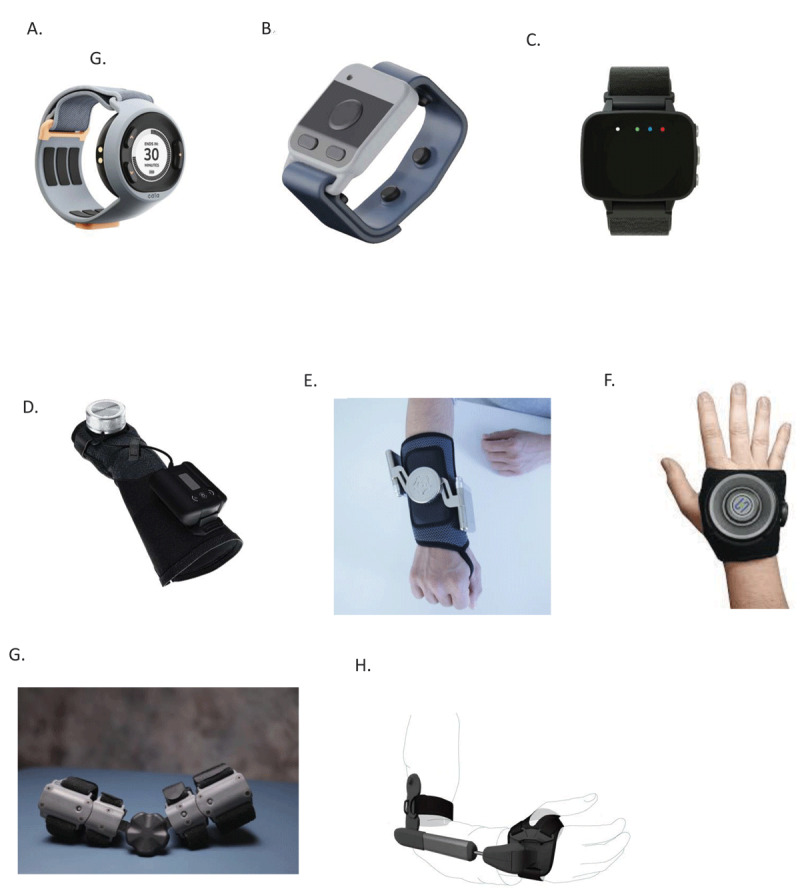
Visual representation of the devices discussed in the review. **A.** Cala Trio, **B.** Encora Plus, **C.** Felix™ NeuroAI™, **D.** GyroGlove™, **E.** Tremelo®, **F.** Steadi-Two®, **G.** Move-D, **H.** STILL orthosis. This figure provides a comparative overview of the various non-pharmacological, non-surgical devices evaluated for their efficacy in reducing action tremor, particularly essential tremor (ET). Each device is visually depicted to illustrate their design and functional components.

The Cala Trio® (Figure 1A) is the best studied and most widely available TAPS device. This device electrically stimulates the radial and medial nerves at the wrist with power settings beneath the motor threshold. It is hypothesized that this stimulation generates afferent potentials to the thalamus, inducing a local field potential that attenuates tremor within the CNS. The device’s stimulation parameters are initially customized to the individual’s tremor frequency, but do not subsequently receive continuous feedback.

Current assessments of the Cala Trio’s efficacy were conducted through one controlled trial and subsequent open-label trials. The PROSPECT trial reported improvements in tremor severity, gauged by accelerometry, and enhancements in daily activities, as measured by the Bain and Findley scale [17]. The initial version, CALA ONE, demonstrated improvements in The Essential Tremor Rating Assessment Scale (TETRAS) scores and hypothetical activities of daily living (ADL) scores, excluding spirals, immediately post-use in a 77-subject sham-controlled trial. Adverse events were minor, mainly skin irritation at the stimulation site.

Subsequent research into the Cala Trio device’s effectiveness reported significant tremor power reduction, as assessed by the device itself, of 71% across sessions, while 65% of participants reported some improved daily functioning [18]. Another open-label study involving 263 patients showed major improvements in tremor severity and daily functioning, with 90% of participants demonstrating some device measured tremor power reduction. Minor device-related adverse events were reported by 18% of patients, but no serious adverse events developed.

There is a finite duration of benefit after stimulation but one report supported improvement for at least 60 minutes post-therapy, with 80% of patients experiencing numeric improvements in clinical ratings [16]. TAPS therapy also has shown benefit when used in conjunction with oral medications, again based on in tremor power and ADL scores [19]. Longer duration open label studies report continued efficacy and safety over prolonged periods, without significant habituation noted after a year of use [20]. A secondary analysis highlighted TAPS therapy’s clinical benefits for ET patients with severe tremors, medication non-responsive tremor, and advanced age [21].

The CALA Trio is commercially available in the U.S. by prescription. It is recommended for use in 40-minute sessions, with anecdotal evidence suggesting tremor improvement lasting 2–3 hours for most users, who often extend device usage beyond the recommended duration. Currently it is approved for only one arm and is available in different sizes depending on wrist circumference [13]. It is covered by U.S. Medicare and variably by commercial insurance.

### 1.3 Fasikl Felix™ Wristband

The Felix™ NeuroAI™ Wristband (Figure 1C), developed by Fasikl, targets tremor through the stimulation of radial, medial and ulnar nerves at the wrist [22]. This device is equipped with tri-axial accelerometry to monitor tremor patterns and provide real-time data for analysis and “personalized” treatment adjustments. This data is then processed by an artificial intelligence system, which analyzes the individual’s tremor amplitude to customize the electrical stimulation current delivered by the wristband. This treatment strategy aims to disrupt oscillatory activity within the cerebellothalamo-cortical circuit. There are multiple sizes, determined by measured distances between the nerves.

Evidence supporting the efficacy of the Felix™ Wristband comes from two prospective studies conducted in the United States and China, though this data has yet been published [23]. Formal sham controlled multi-center clinical trials are ongoing in North America and China to evaluate the safety and effectiveness of the Felix™ Wristband [24]. It is not yet commercially available.

### 1.4 Motimove®

The Motimove® system, developed by 3F-Fit Fabricando Faber in Serbia, is a therapeutic device that employs multiple stimulators to activate antagonistic forearm muscles through out-of-phase stimulation. This approach delivers electrical current pulses directly to the flexor and extensor muscles, “triggering the depolarization of motor neurons”. This action counteracts the tremorgenic activity via activation of antagonistic muscles in the forearm. While the precise muscles targeted can vary based on individual patient needs, they generally include key flexor and extensor groups such as the Flexor Carpi Radialis, Flexor Carpi Ulnaris, Extensor Carpi Radialis, and Extensor Digitorum, among others.

The system consists of a multi-channel electronic stimulator that communicates via Bluetooth with a remote computer. This setup allows for the selection of applications, fast one-time calibration, and the setup of stimulation parameters, as well as sensor-driven control. The inclusion of inertial measurement units (IMUs) within the MotiMove® system, which combine accelerometer and gyroscope sensors, enables real-time feedback and adjustment of stimulation. A small open label study on the Motimove® system demonstrated efficacy, with a 67% reduction in the amplitude of tremor observed in six out of seven patients affected by ET or PD [25,26]. The Motimove® received a CE marking for use in the European Union, but to our knowledge, the device is not widely available [27].

## 2. Orthotic Devices that Physically Dampen Movement

Orthotic devices fall into two main categories: active orthotics, which include technologies like WOTAS (Wearable Orthosis for Tremor Assessment and Suppression) and exoskeletons, and passive/semi-active orthotics, such as the GyroGlove™, Tremelo™, Steadi-One, and Readi-Steady™. Active orthotics detect tremor in real-time and respond with more precise counter-movements or adjustments. These devices often incorporate sensors and actuators, providing dynamic, responsive support tailored to the individual’s movements.

Passive and semi-active orthotics leverage mechanical principles to dampen tremor without the need for electronic feedback mechanisms. Devices like the GyroGlove™ use gyroscopic action to stabilize the hand, while others, such as the Tremelo™ and Steadi-One™, employ counterweights or fluid dynamics to absorb and neutralize the force of involuntary movements.

### 2.1 Active Orthotic Device

#### 2.1.1 Wearable Orthosis for Tremor Assessment and Suppression

The Wearable Orthosis for Tremor Assessment and Suppression (WOTAS) device uses robotic exoskeleton mechanisms. WOTAS reportedly differentiates between voluntary movements and tremors, employing advanced algorithms to identify tremor movements specifically. The devices employ two principal strategies. Impedance control dynamically adjusts the interaction between the device and the limb, altering the perceived elasticity and damping. Repetitive control actually applies counter-forces generated by DC motors as actuators to oppose the direction of involuntary movement [28,29].

A small clinical evaluation of WOTAS yielded encouraging outcomes, demonstrating its capacity to significantly lessen tremor amplitude [30]. Exoskeleton achieved an average of 40% reduction in tremor power across all users, with a potential of up to an 80% reduction in specific cases, primarily measured using gyroscopes to assess changes in tremor amplitude within the frequency band of 2 to 8 Hz through power spectral density analysis [30].

However, the device faces challenges such as refining the user-device interface for better comfort and force application and accommodating the broad variability in tremor characteristics across patients, necessitating extensive customization; these factors contribute to the current absence of such devices from the market, highlighting the need for further ergonomic designs to ensure practicality and user acceptance [31].

### 2.2 Passive/Semi-Active Orthotic Devices

#### 2.2.1 Magnetorheological Fluid-Based Exoskeleton System

Another type of active orthotic device is an exoskeleton system for tremor suppression that utilizes magnetorheological (MR) fluid. This system, designed at School of Mechanical Engineering at Shanghai Jiao Tong University, China is designed to provide real-time adjustable damping force for wrist tremor suppression [32].

The MR fluid within the device responds to magnetic fields, enabling dynamic adjustment of the fluid’s viscosity and thus the damping force exerted by the system. Sensors, including accelerometers, gyroscopes, and EMG sensors, detect movements and tremor, providing data that the system uses to modify the magnetic field accordingly. This mechanism enables the exoskeleton to adapt to the severity and frequency of the wearer’s tremors in real-time.

Data from preliminary mechanical testing indicates that the system can effectively reduce the amplitude of acceleration (60.4%) and angular velocity (55%). There is no human data.

Future work aims to further optimize the device’s performance through design improvements and conduct more extensive testing on patients to validate its efficacy in real-world applications. Other electromagnetic based braces and fluid based dampeners are also under development [33].

#### 2.2.2 GyroGlove™

The GyroGlove™ leverages gyroscopic stabilization principles to counter involuntary hand movements. Gyroscopes utilize the principles of angular momentum and gyroscopic precession to counteract acceleration. A gyroscope within the glove spins at high speeds up to 10,000 rev/minute, creating significant angular momentum, which is a function of the rotation velocity, mass, and shape. This angular momentum enables the gyroscope to maintain its orientation, making it resistant to changes in direction orthogonal to the plane of the angular momentum [34].

When a hand tremor introduces an external force, it attempts to alter the gyroscope’s orientation. However, due to the conservation of angular momentum, the gyroscope, attached to a tight fitting glove resists these changes. Instead of moving in the direction of the applied force, the gyroscope undergoes gyroscopic precession, moving at a right angle to the force. This movement generates a stabilizing force that counteracts the tremor’s motion, providing a corrective torque that opposes the unwanted movement [35]. For tremor suppression, the ideal gyroscope would be fairly small and light weight, but spin as rapidly as possible, and be as close to the wrist as possible [36].

Impedance control dynamically adjusts the interaction between the device and the limb, altering the perceived elasticity and damping. Repetitive control actually applies counter-forces generated by DC motors as actuators to oppose the direction of involuntary movement [28,29].

This controlled trial aims to quantify the device’s ability to improve tremor severity and functional capacity in a clinical setting [37].

The GyroGlove™ is somewhat bulky and may be difficult for some to wear continuously. Furthermore, the device’s effectiveness may vary among individuals, with not all experiencing the same level of tremor control, mostly as a function of tremor direction. Theoretically, as configured, it best dampens pronation and supination oscillation around the wrist. The device is recently available for cash purchase but is expensive.

#### 2.2.3 Tremelo™

The Tremelo™ device is a tightly fitting wearable glove that fits around the hand and arm, attached to two tuned mass dampeners (TMD) (Figure 1E). TMD technology, often used in structural engineering to reduce vibrations in buildings and bridges, has been adapted in the Tremelo™ device to counteract the oscillations caused by ET. The principle behind a TMD is relatively straightforward: it consists of a mass that is tuned to oscillate out of phase with the unwanted parent oscillation (in this case, tremor). The device uses two counterbalance weights in different planes. By doing so, it absorbs and dissipates the energy of the tremor, reducing their amplitude [38]. The main advantage of TMD is that being a mechanical solution, it does not require electrical power, enhancing its ease of use and maintenance.

The Tremelo™ device, according to Five Microns, reduces essential tremor by 85–90%, but this claim is not supported by published clinical data, and the methodology for this effectiveness measure is not specified. In our experience, some users find the device heavy and cumbersome, which may lead to discomfort or fatigue with long-term wear. The device is available in the United States without a prescription but is expensive.

#### 2.2.4 Steadi-Two

The Steadi-Two (Steadiwear) (Figure 1F) is an assistive glove designed to provide stabilization of hand tremor. It employs a ball-joint surrounded by a “non-Newtonian smart fluid”, which allows for fluid movement under normal conditions, but which instantly increases in viscosity in response to tremor, providing targeted stabilization. The non-Newtonian fluid is located within a chamber that encompasses the ball-joint. This fluid remains easily flowable under normal conditions, allowing for the natural movement of the hand. However, upon experiencing the sudden movements characteristic of tremor, the fluid quickly solidifies, providing immediate resistance.

Per the manufacture, the ball-joint mechanism, encased in this non-Newtonian fluid is critical for distinguishing between voluntary movements and involuntary tremor. During intentional hand movements, the glove permits fluid motion without resistance. Conversely, when tremors occur, the rapid motion causes the fluid to harden around the ball-joint, creating an immediate counterforce that dampens movement in the hand [39]. There is no published clinical trial data.

Currently the second generation Steadi-Two, is available for purchase without a prescription. Cost is moderately expensive.

#### 2.2.5 Readi-Steadi®

The Readi-Steadi® Anti-Tremor Orthotic Glove System customizable glove is marketed for both ET and PD hand tremor. The system features a snug-fitting glove as its foundation, to which various custom orthotic components such as straps, weights, and supports are added, depending on the user’s specific tremor pattern.

The product utilizes a strategic weight distribution to mitigate hand tremors by applying gentle pressure on targeted areas of the hand and arm. These areas are selected based on a visual analysis of the user’s specific tremor characteristics as determined by a video submitted to the manufacturer. The glove superficially resembles a regular glove.

The addition of weights to the glove acts as a counterbalance that reduces the amplitude of action tremors by stabilizing the hand. It is proposed to do so not by pushing against the tremor or pulling in the opposite direction, but through enhancing proprioceptive feedback and motor control, thereby improving precision [39]. There is no published clinical data and no attempted clinical trials.

The Readi-Steadi® Anti-Tremor Orthotic Glove System requires a physician order and may be covered by insurance.

#### 2.2.6 Move-D Orthotic Brace

The Move-D orthotic brace, developed by a group in California, applies adjustable tension that resists movement around the elbow joint. It does not impede any other joint, so would not be expected to improve tremor oscillating around the wrist or fingers. One small open label pediatric study of subjects with mixed tremor types did not show any significant improvement in functional abilities and did not assess any tremor scales, but 5/10 subjects felt there was some improvement [40]. The device is for sale in the U.S. without a prescription.

#### 2.2.7 STIL Orthosis

The STIL orthosis is another dampening brace that attaches just above the elbow and around the palm of the hand and could potentially dampen tremors oscillating around the elbow and wrist. There is no published efficacy data. The device was developed in Holland and is anticipated to be for sale in the near future.

In conclusion, there are numerous devices, which using various techniques to assess the involuntary tremor movement, physically dampen tremor. Challenges remain to differentiate volitional movement from tremor, adapt to the constant changes in tremor direction, to physically restrain tremor without physical discomfort, and to be less invasive and more practical.

## 3. Limb Cooling

Limb cooling as a therapeutic intervention for tremor management has shown promise in various contexts, including ET, multiple sclerosis (MS), and drug-induced tremors [41,42]. This approach is grounded in the premise that cooling of the limbs can modulate neural and muscular activities, thereby reducing tremor amplitude and frequency. Limb cooling is shown to decrease both motor and sensory conduction velocities of the ulnar nerve immediately after deep and moderate cooling interventions [43]. The sensory nerve conduction velocity returns to normal 20 minutes post-cooling, while the motor nerve conduction velocity remains diminished, indicating a sustained effect of cooling on motor nerve function.

A study focusing on patients with multiple sclerosis demonstrated that both deep and moderate cooling of the tremor-affected arms reduced tremor amplitude and frequency without adversely affecting heart rate or central body temperature [44]. The study used cryomanchets (cold packs) for forearm cooling and multiple cold packs for whole arm cooling, with protective measures against skin damage, to achieve limb cooling over a 15-minute period [44]. Limb cooling also reduces tremor amplitude, and both motor and sensory conduction velocities of the ulnar nerve, in tacrolimus-induced tremors, a common side effect in transplant recipients [41].

Currently there are no commercial products specifically designed for this purpose, but these studies underscore the potential of limb cooling as an easy and inexpensive intervention for tremor management across different patient populations. The mechanisms of action, including the impact on nerve conduction velocity and muscle spindle activity, suggests cooling could improve multiple tremor etiologies, and possibly other hyperkinetic disorders like dystonia. Future research and development efforts should focus on creating specialized cooling devices that are practical for everyday use.

## 4. Vibration therapy

Vibration therapies, which have been used therapeutically since the time of Charcot, produce rapid oscillatory motions that are applied to the body or specific parts of the body [45]. There are several mechanisms through which vibration therapy is proposed to modulate nervous system activity, though it’s important to note that the scientific understanding of these processes is evolving, with varying degrees of empirical support. Vibration therapy stimulates mechanoreceptors in the skin and muscles, reach the thalamus via the cuneate nucleus and can potentially alter central sensory and motor processing, potentially “distracting” or “overriding” the neural signals associated with tremor [27]. A gating mechanism theory, similar to the gate control theory of pain, posits that vibratory inputs might modulate neural circuit activity, thereby potentially reducing tremor amplitude through a process of spinal or cerebral gating. Moreover, vibration may improve motor control and coordination by bolstering proprioceptive feedback, crucial for limb stabilization and involuntary movement reduction. It impacts motor areas in the cortex and cerebellum, potentially mitigating the excessive neural output associated with tremor generation [46]. Additionally, relaxing muscles and decreasing stiffness may directly reduce muscle contraction.

### 4.1 Encora Pulse™

The Encora Pulse™ is a wrist worn vibration device with 4 contact points in the lateral and volar wrist that is being developed for ET and PD tremor (Figure 1B). The device synchronizes vibration frequency with the peak tremor frequency, and vibrates with a 2 mm amplitude. An unpublished 9 subject open-label trial reported a mean 50% reduction in accelerometry based tremor power while the device was active. A separate unpublished 8 subject dataset found a significant 0.5pt improvement in TETRAS spirals compared to a sham group. A larger sham- controlled trials has also been completed but not presented. (ClinicalTrials.gov ID NCT06343285) Anecdotally, tremor returns when the vibration stops. The device is not yet available for purchase.

### 4.2 Vilim ball™

The Vilim ball is a compact, smooth spherical device suitable for one-handed use. The individual holds it in their hand and ball vibrates at 8–18 Hz, with an amplitude of 0–2 mm. Vilim ball therapy might provide temporary relief from symptoms for some individuals [47]. More robust, large-scale research is needed to conclusively establish their efficacy, optimal usage parameters (e.g., frequency and duration of use), and long-term benefits [48]. One obvious issue is that the hand can’t be used for anything else while holding the ball, and it is not clear if the proposed tremor reduction persists after use. The Vilim ball is licensed as a class 2a medical devise and available for purchase without prescription. Cost is moderate.

## 5. Adaptive utensils

**Figure 2:**
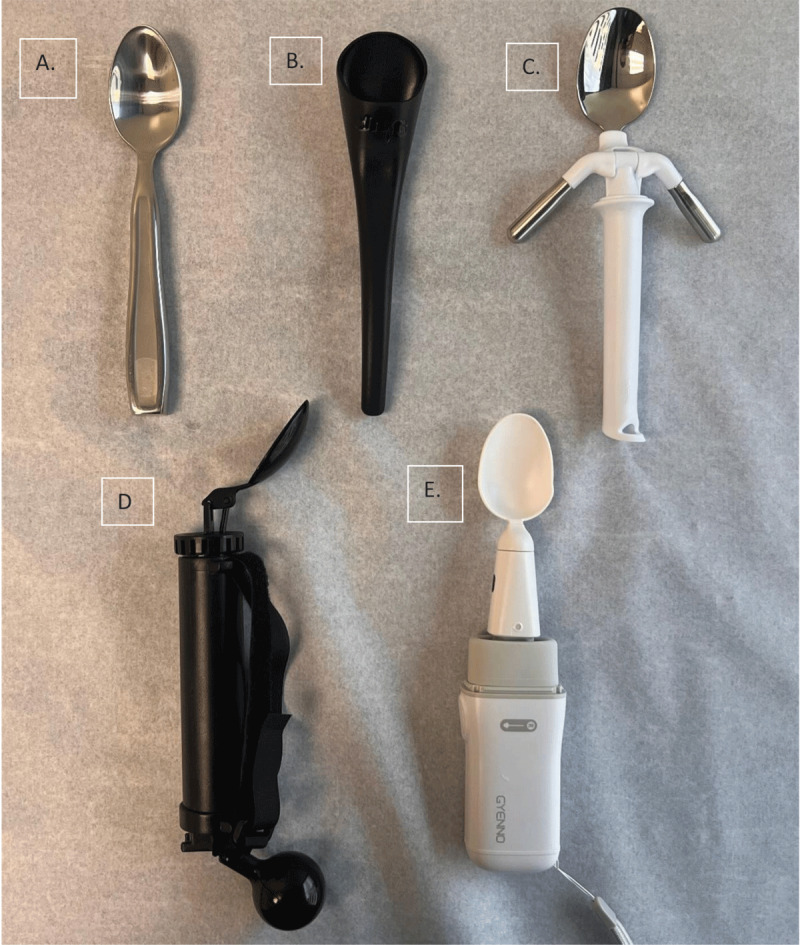
Visual representation of the adaptive utensils discussed in the review. **A.** Weighted Spoon, **B.** S’Up Spoon, **C.** ELISpoon™, **D.** Steady Spoon™, **E.** GYENNO Spoon II. This figure showcases various adaptive utensils designed to mitigate the impact of tremor on daily activities, particularly eating. Each utensil is illustrated to demonstrate their unique design features and functional mechanisms aimed at improving the quality of life for individuals with essential tremor (ET) and other action tremors.

Adaptive utensils do not alter the patient’s actual tremor but lessen the functional impact of the tremor on that specific task, usually eating. The range of available adaptive utensils is broad (Figure 2). Simple ergonomic improvements such as a deeper cavity can reduce spillage. Simple weighted utensils add stability and may reduce the amplitude of tremors through added mass. Counter-weighted utensils possible reduce tremor amplitude at the end of the utensil by pivoting the device based on a position dependent weight. Electrical devices use sensors and actuators to move the utensil tip opposite to the direction of the tremor movement. The development of dishes with non-slip bases and utensils with non-slip handles may further support eating.

### 5.1 Weighted Spoons

Weighted spoons may improve functional eating by leveraging principles of inertia (Figure 2A). The added mass of these utensils increases their inertia, making them initially more resistant to the involuntary alternating acceleration movements caused by tremor, enabling users to eat with greater ease. The increased mass increases the force needed to change movement direction, potentially disperses the kinetic energy from the tremor, reducing the amplitude of the movement transferred to the utensil [49].

There are many weighted spoons available on the market which come in various weights, sizes, and grips, allowing individuals to choose the most comfortable and effective option for their specific condition. One significant advantage of weighted spoons is their ability to be used discreetly in most social situations, unlike many other assistive devices. However, for some individuals, the added weight might be too much for their specific level of strength or dexterity, potentially leading to fatigue over prolonged use. Additionally, finding the right weighted spoon that provides the best balance of control and comfort might require testing several different models, which can be time-consuming and increase cost [50].

There is no published clinical trial data on weighted spoons for ET. They are inexpensive and widely available without prescription.

### 5.2 S’Up™

The S’Up Spoon was conceived and developed by an individual with cerebral palsy to aid those who find it challenging to eat due to tremors, limited hand mobility, or coordination issues (Figure 2B). The S’Up Spoon has a deep cavity that securely holds food, such as soup or cereal, designed to reduce spills. This may be especially beneficial for people with conditions like ET or any other condition that might cause involuntary hand movements [51].

There is no published data of efficacy in ET, however in our clinic this simple and inexpensive device was often the preferred adaptive device when tested. It is available for sale without prescription and is inexpensive.

### 5.3 Weighted Counterbalance

#### 5.3.1 ELISpoon™

The ELISpoon is an assistive device, designed to stabilize the spoon cavity during eating by using counterweights and rotation axes (Figure 2C). Advantages of counter weighted devices include the lack of required maintenance or internal power source. The ELISpoon has two counterweights near the spoon cavity, which is attached via a ball joint to the spoon handle. While it seeks to reduce spillage and facilitate independent eating, feedback suggests that its performance may vary with the user’s tremor intensity and direction. Most users in our clinic reported that the counterweights can obstruct the spoon cavity when brought towards the mouth making it difficult to use.

#### 5.3.2 Steady Spoon™

The Steady Spoon features a counterweight mechanism placed in the handle (back) to stabilize the utensil, potentially reducing spillage for individuals with hand tremor (Figure 2D). This design innovation results in less movement of the end of the spoon compared to the held handle. The utensil is further enhanced by a built-up handle, equipped with a hook & loop strap, ensuring a secure grip without necessitating a strong grasp. However, the spoon’s smaller size and the directional limitation of the counterweight’s effectiveness in the handle may not fully meet the diverse needs of many users, as the spoon cavity does not rotate with the handle when patients try to poor the content into their mouths, limiting usefulness.

### 5.4 Electrical actuators

#### 5.4.1 GYENNO™

The GYENNO Spoon II is designed for individuals with PD and ET (Figure 2E). It is compact, with a spoon suitable for liquids and a fork attachment for varied eating needs. The spoon actuators trigger off a gyroscopic mechanism to counter-act both vertical and horizontal movements and are adaptable for left or right-handed use, adjusting the level of control based on the eating action. It uses an electrical actuator motor to move the spoon cavity opposite of that tremor induced movement in the vertical, lateral and rotation planes. It comes equipped with WiFi and a built-in sensor for motion tracking, aiding in the collection of data for movement disorder research. People can download an app and simulate eating with their phone to “test” if the device might help their tremor prior to purchase, however it is not reported what parameters constitute a successful test. The device is portable, with an internal battery, eliminating the need for a charging station and supporting mobility and independence during meals. One small study did not show benefit in patients with Parkinson’ disease but there are no clinical trials or published data in ET [52]. Cost is moderate and does not require a prescription.

#### 5.4.2 Liftware Steady™

The Liftware Steady is an assistive device marketed for PD or ET. It consists of an electronic handle equipped with accelerometry sensors and actuator motors, which together actively counteract the movements of hand tremor. The device comes with various attachments, such as a soup spoon, fork, and spork. Its functionality hinges on detecting the user’s tremors and then moving the attached utensil in the opposite direction to stabilize it, reportedly reducing tremor effects by up to 70% during use per the manufacturer website. There is no published data. Cost is moderate and does not require a prescription.

## Summary

In summary, non-pharmacological, non-surgical interventions for action tremor, particularly in ET, include transcutaneous peripheral nerve stimulation (PNS), orthotic/mechanical devices, cooling and vibration strategies, and adaptive utensils. The PNS technologies, including open loop (CALA-Trio) and closed loop systems (Felix™ NeuroAI™ and Motimove®), along with orthotic devices such as Tremulo™ and GyroGlove™ offer potential options yet mostly lack published clinical trial data. While many of these non-invasive devices are available, there remains a significant need for controlled clinical trials to confirm their efficacy. Thus, further research is essential to establish the therapeutic value of these interventions in managing ET and other action tremors.
